# Influence of local strain caused by cycloaddition on the band gap control of functionalized single-walled carbon nanotubes†

**DOI:** 10.1039/c9ra02183c

**Published:** 2019-05-08

**Authors:** Yutaka Maeda, Kiyonori Kuroda, Haruto Tambo, Hiyori Murakoshi, Yui Konno, Michio Yamada, Pei Zhao, Xiang Zhao, Shigeru Nagase, Masahiro Ehara

**Affiliations:** Department of Chemistry, Tokyo Gakugei University Tokyo 184-8501 Japan ymaeda@u-gakugei.ac.jp; Research Center for Computational Science, Institute for Molecular Science Okazaki 444-8585 Japan ehara@ims.ac.jp; Institute for Chemical Physics, Department of Chemistry, School of Science, State Key Laboratory of Electrical Insulation and Power Equipment, Xi'an Jiaotong University Xi'an 710049 China; Fukui Institute for Fundamental Chemistry (FIFC), Kyoto University Sakyou-ku Kyoto 606-8103 Japan

## Abstract

Fine control of the band gap of single-walled carbon nanotubes (SWNTs) has been achieved by the functionalization with dibromoalkanes, namely, 1,3-dibromopropane (1a), 1,4-dibromobutane (1b), 1,5-dibromopentane (1c), and 1,8-bis(bromomethyl)naphthalene (1d). Red-shifted photoluminescence (PL) peaks observed at 1215–1242 nm were assigned to the local band gaps of the chemically functionalized SWNTs 2a, 2b, 2c, and 2d, respectively. Density functional theory (DFT) and time-dependent DFT calculations for 2a–2d suggest that “local strain” induced by cycloaddition plays an important role in tuning the local band gap energies of functionalized SWNTs.

## Abstract 2

The local strain at the addition is cited as another factor controlling the emission wavelength of functionalized SWNTs.

## Graphical abstract

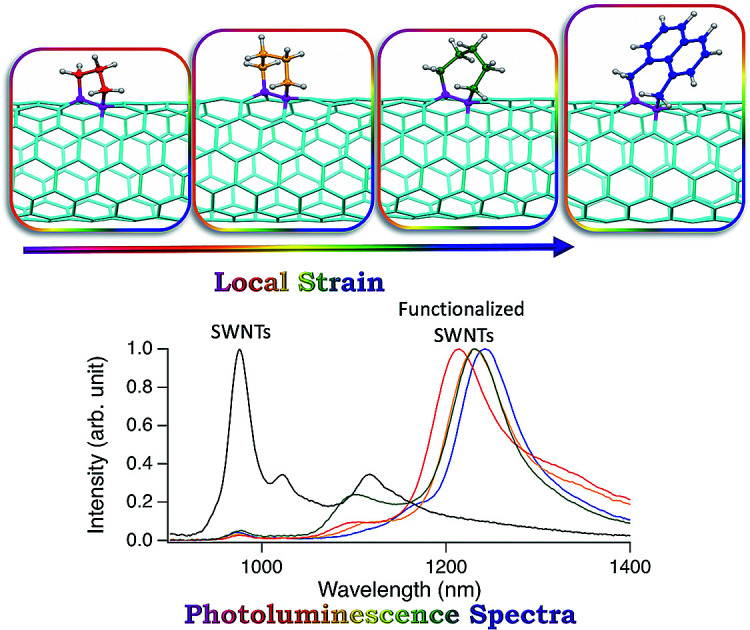

## Introduction

Single-walled carbon nanotubes (SWNTs) have attracted significant interest due to their remarkable mechanical stability and intrinsic optical and electrical properties.^1,2^ Their various electronic states based on the diameter and helicity make SWNTs attractive as the building blocks of electronic and optical nanodevices. In 2002, near-infrared (NIR) photoluminescence (PL) of semiconducting SWNTs was observed in individually dispersed SWNTs.^3^ PL measurements of SWNTs have been used as a useful analytical method to assign SWNT structures because the first (*E*_11_) and second (*E*_22_) optical transition energies can be identified from the excitation and emission energies. As NIR photoluminescent materials, SWNTs are expected to be of practical use in the imaging of biological tissues, telecommunications, and other applications.^4–8^ Recently, it has been reported that the chemical functionalization of SWNTs, such as photooxidation, arylation, and alkylation, induces bright and red-shifted NIR PL peaks.^9–19^ Based on density functional theory (DFT) calculations, it was proposed that the new PL peak is derived from splitting of the frontier orbitals of SWNTs by sidewall functionalization.^9^ Miyauchi *et al.* reported that the PL quantum yield of SWNTs (typically ∼1%) was increased by at least one order of magnitude (to ∼18%) by oxidation at a moderate functionalization degree.^10^

Wang *et al.* reported the observation of new PL peaks in the range of 1110–1148 nm (PL) from arylated (6,5) SWNTs depending on the substituents of the phenyl group. A good correlation was found between this emission wavelength and Hammett's constant, which revealed that the electronic effect of the substituent is one of the factors in emission wavelength control.^13^ Furthermore, Wang *et al.* reported that the emission wavelength can be controlled by the number of fluorine atoms in fluoroalkylated SWNTs.^16^ Recently, we reported that the dialkylation of (6,5) SWNTs using butyllithium and butyl bromide and their subsequent thermal treatment resulted in a new strong PL peak at around 1220 nm (
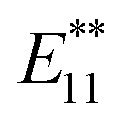
 PL).^13^ Additionally, (6,5) SWNTs functionalized using 1,2-bis(bromomethyl)benzene predominantly resulted in a new PL peak at 1231 nm.^15^ These results indicate that controlling the distance of the addition site carbons on the SWNTs is another factor that controls the emission wavelength. The contribution of the addition site carbons on the difunctionalized SWNTs has been confirmed theoretically by Htoon *et al.*^20,21^

In this work, we focus on local strain at the addition cites as another factor controlling the emission wavelength of functionalized SWNTs. Yang *et al.* reported theoretical studies showing that the electronic states of pristine SWNTs varied with strain and that the changes in the band gap depend on their chirality and deformation mode.^22,23^ Pioneering experimental work on tuning the electronic properties of pristine SWNTs with mechanical strain by monitoring the change in band gap and PL in response to applied strain have also been reported.^24–26^ The introduction of a cyclic addenda on the SWNT can be expected to produce local strain depending on the alkyl chain length. In addition, changes in the local band gap energy can be measured with high sensitivity by PL measurements. In the present study, we functionalized SWNTs using dibromoalkanes, 1a–1d, to clarify the influence of the local strain induced by cyclic addenda on the red-shifted PL wavelength of functionalized SWNTs. These dibromoalkanes have been used for the synthesis of cyclization products of fullerene C_60_.^27,28^

## Experimental

### General information

The (6,5)-enriched SWNTs (SG 65i), used in this work, were purchased from Sigma-Aldrich. Reagent-grade dibromoalkanes were purchased from commercial suppliers. 1d was synthesized according to literature procedure.^29^ Optical absorption spectra were recorded by using a spectrophotometer (V-670; Jasco Corp.) equipped with a Pyrex cell with a 10 mm path length. In addition, Raman spectra were measured under excitation at 514.5, 561, and 633 nm, by using a spectrophotometer (LabRAM HR-800; Horiba Ltd.). These spectra were normalized relative to the G band. Photoluminescence spectra were obtained by using a spectrophotometer equipped with a 450 W lamp and a Symphony-II CCD detector (Nanolog; Horiba Ltd.). The excitation and emission wavelengths were varied from 500–1000 nm and 827–1400 nm, respectively, in 1 nm steps. The excitation and emission slit widths were 10 nm. The PL intensity was corrected to the data correction time of each sample. For absorption and PL measurements, the samples were dispersed in D_2_O containing 1 wt% sodium dodecylbenzene sulfate (SDBS) by ultrasound irradiation in a bath sonicator (B2510J-MT ultrasonic cleaner; Branson) and centrifuged at 140 000*g* for 1 h in a high-speed centrifuge equipped with a P70AT2 angle rotor (CP80β; Hitachi Koki Co., Ltd.). To adjust the absorption intensity near 775 nm, an adequate dose of D_2_O solution containing SDBS was added to the dispersion depending on the concentration. After sonication, the resulting suspension was centrifuged for 1 h at 140000*g*. Scanning electron microscopy (SEM) was conducted using a field emission electron microscope (1.5 kV accelerating voltage, 10 μA beam current, SU8020; Hitachi Ltd.). Thermogravimetric analysis (TGA) of the samples was performed at a heating rate of 10 °C min^−1^ and a gas (N_2_ or air) flow rate of 50 mL min^−1^ (TG-50A; Shimadzu Corp.).

### Typical procedure of reductive alkylation^30^

Naphthalene (152 mg, 1.19 mmol) and sodium (77.8 mg, 3.38 mmol) were placed in a 200 mL heat-dried three-necked round-bottomed flask under argon. Anhydrous tetrahydrofuran (50 mL) was then added to the flask and the contents were stirred for 1.5 h. A portion (5.0 mg) of SWNTs was placed in a second 200 mL heat-dried three-necked round-bottom flask under argon and the sodium naphthalenide solution was added to the SWNTs, and the mixture was then sonicated for 1 h. Dibromoalkane (1.43 mmol) was subsequently added to the mixture, which was sonicated for 30 min. After the addition of dry ethanol (15 mL), the resulting suspended black solid was collected by filtration using a membrane filter (PTFE, 1.0 μm) and washed with tetrahydrofuran, methanol, and distilled water by the dispersion/filtration process. The resulting films were analyzed with Raman spectroscopy and used for preparation of SWNT dispersion.

### Computational details

A single unit of pristine (6,5) SWNT passivated with hydrogen at the terminals was utilized as the computational model. SWNT-C_3_H_6_, SWNT-C_4_H_8_, SWNT-C_5_H_10_, and SWNT-C_12_H_10_ adducts were calculated at the *ortho* and *para* positions in three different directions (L_−33_, L_87_, L_27_), as shown in Fig. 2. The ground state of these derivatives was optimized by DFT using the B3LYP functional with 6-31G*.^31–33^ The TD-DFT calculations of the vertical transition energies and the optimization of excited states were performed at the B3LYP/3-21G level of theory. To evaluate the effects of the distortion of the nanotube and π-conjugation of the C_12_H_10_ adduct on the transition energy, we also examined SWNT-(CH_3_)_2_ with the same nanotube structure as SWNT-C_12_H_10_. Acyclic addenda were also examined in the same manner. All DFT calculations were conducted using the Gaussian 09 suite of programs version E.01.^34^

## Results and discussion

**Scheme 1:**
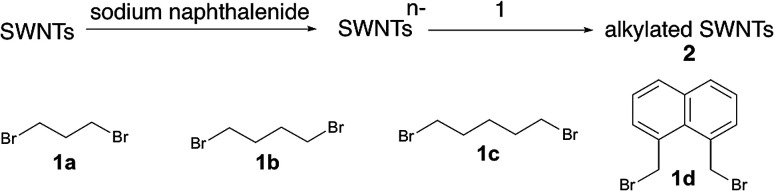


**Fig. 1:**
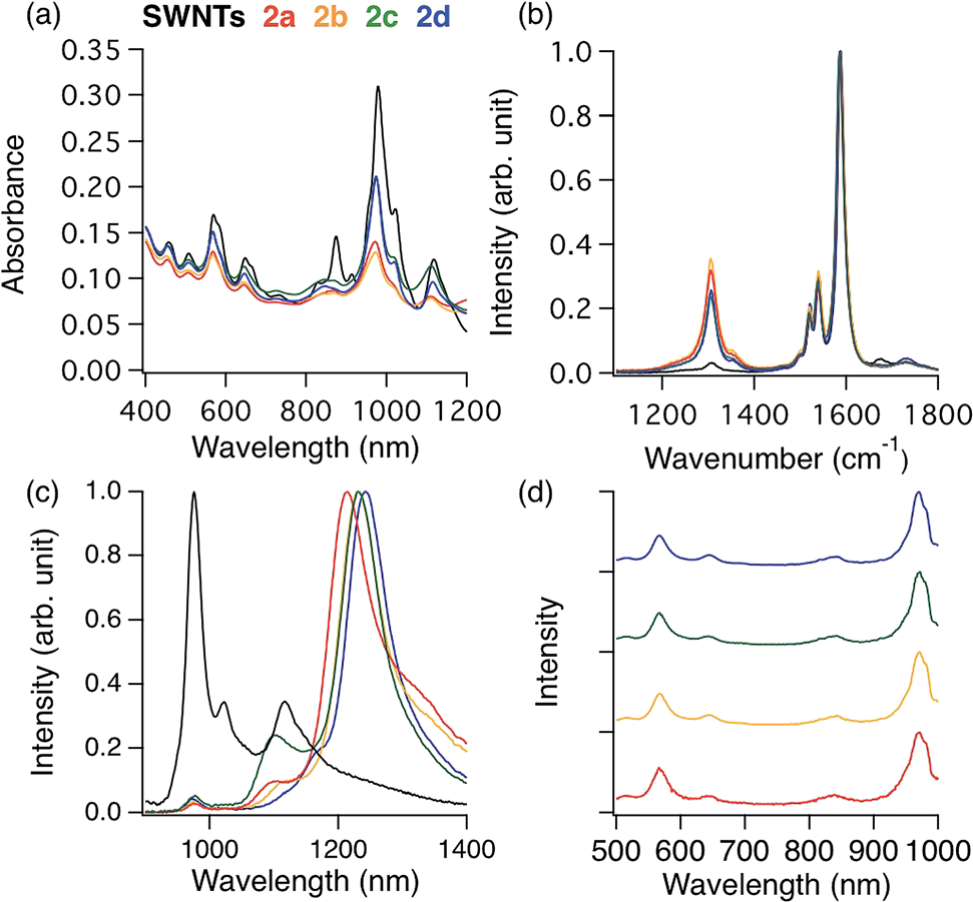
(a) Absorption spectra of 2 dispersed in a D_2_O solution containing 1 wt% SDBS. (b) Raman spectra of 2 (film) excited at 561 nm. Black: SWNTs. (c) Normalized PL spectra excited at 567 nm of 2 dispersed in a D_2_O solution containing 1 wt% SDBS. (d) Normalized excitation spectra of 2. Red: 2a, 1215 nm. Yellow: 2b, 1230 nm. Green: 2c, 1231 nm. Blue: 2d, 1242 nm.

The alkylation of SWNTs was conducted using commercially available (6,5) SWNT-enriched SWNTs, sodium naphthalenide, and dibromoalkanes (1), as shown in Scheme 1.^15,30^ The functionalization degree of the alkylated SWNTs (2) was evaluated by the characteristic absorption peak intensity at ∼980 nm (*E*_11_) and the intensity ratio of the D band to the G band (D/G ratio: [D/G]) in the Raman spectra (Fig. 1).^35^ The *E*_11_ abs. ratio is used as an index for the degree of functionalization of SWNTs, and is defined by the relative peak intensity, [*E*_11_ absorption intensity of 2/intensity of local minimum near 775 nm of 2]/[*E*_11_ absorption intensity of SWNTs/intensity of local minimum near 775 nm of SWNTs];1
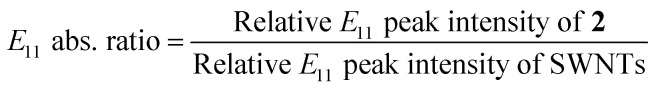


**Fig. 2:**
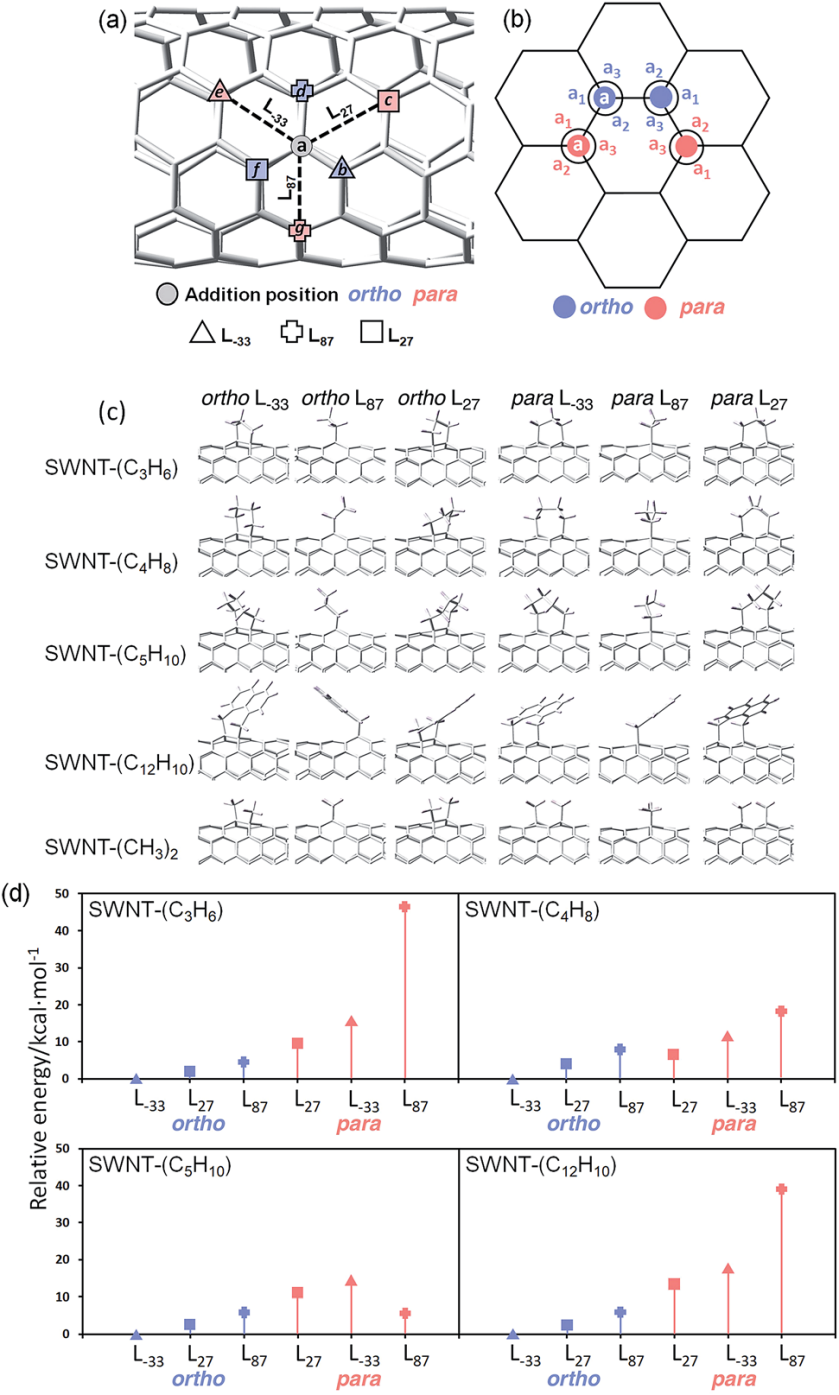
(a) Three different directions of *ortho* and *para* addition sites in (6,5) SWNTs. The italicized letters (*a*, *b*, *c*, *d*, *e*, *f*, and *g*) represent different carbon atoms. (b) Representation of three bond angles (a_1_, a_2_, a_3_) in *ortho* and *para* adducts (the italicized letter “*a*” represents the addition site as shown in Fig. 2a, and the other unlabeled addition site represents “*b*, *d*, or *f*” for *ortho* adducts and “*c*, *e*, or *g*” for *para* adducts). (c) The optimized partial structures of different substituents in functionalized (6,5) SWNTs. (d) Relative energies (in kcal mol^−1^) of functionalized (6,5) SWNTs calculated by DFT with B3LYP/6-31G*.

The local minimum at ∼775 nm was selected for the normalization because there is no characteristic absorption peak at that wavelength, and the absorbance of the characteristic peaks changed only in the photoreaction of SWNTs with aliphatic amines.^36^ As shown in Table 1, higher D/G values and a larger decrease of the *E*_11_ abs. ratio were observed for 2a and 2b than for 2c and 2d, indicating that 2a and 2b have higher functionalization degree than 2c and 2d. It is well known that in the cyclization reaction of alkenyl radicals, the yields increase in the order of the formation of 7-, 6-, and 5-membered rings.^37^ The high functionalization degrees of 2a and 2b are consistent with the typical radical cyclization (Scheme 1).

**Table 1:** *E*_11_ abs. ratio, D/G, and 
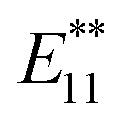
 PL peak wavelength of 2

SWNTs	*E*_11_ abs. ratio	D/G			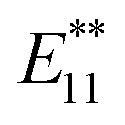 PL peak
		514.5 nm	561 nm	633 nm	
2a	0.45	0.44	0.32	0.40	1215 nm
2b	0.40	0.46	0.36	0.35	1230 nm
2c	0.61	0.28	0.24	0.23	1231 nm
2d	0.66	0.28	0.26	0.24	1242 nm

Fig. 1c shows the PL spectra of 2a–2d normalized to the 
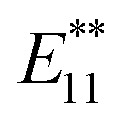
 PL peaks. 
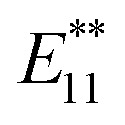
 PL peaks were observed in the spectra of 2a–2d, as dominant peaks, at 1215, 1230, 1231, and 1242 nm, respectively. The excitation spectra of these emissions are in good agreement with the absorption spectra of (6, 5) SWNTs, indicating that the PL peaks assigned to the functionalized (6, 5) SWNTs (Fig. 1d). When bromoalkane was used for the reaction, an 
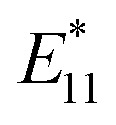
 PL peak at approximately 1100 nm and an 
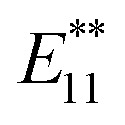
 PL peak emerged after functionalization.^15^ Independently, Shiraki and Nakashima *et al.* reported that two new PL peaks at 1129 and 1253–1258 nm emerged in the reaction of SWNTs with reagents having two reactive benzene diazonium moieties.^17^ The selective emergence of the 
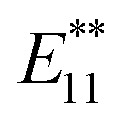
 PL peaks in 2a–2d, similar to that in SWNTs functionalized using 1,2-bis(bromomethyl)benzene, indicates that a highly selective cyclization addition was proceeded in the reaction of SWNTs with 1a–1d.^15^

It is noteworthy that the 
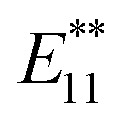
 PL peaks in 2a–2d were observed to vary in the range of 1215–1242 nm upon changing the alkyl chain length. Wang *et al.* reported that the emission energy of functionalized (6,5) SWNTs by mono-substituted phenyl group can be controlled between 1110 and 1148 nm depending on the introduced electron withdrawing and electron donating groups in the phenyl groups.^12^ The difference in the electronic effects of 1a–1c is presumed to be small because difference of 1a–1c is the number of methylene units. It was reported that the wavenumber of Raman peaks is sensitive to the doping and mechanical strain of SWNTs.^38–40^ No significant difference of the wavenumber corresponding to the red shifted PL peaks was observed for 2a–2d (Fig. S2†), supporting that the addenda influence locally at the addition sites. Previously, we reported the PL properties of butylated SWNTs having different functionalization degree.^41^ Two butylated SWNTs have a large difference in the functionalization degree, but a small difference in new red-shifted PL wavelength (Fig. S5†). In contrast, 2a–2d have a small difference in the functionalization degree compared to butylated SWNTs, but a large difference in the PL wavelength, suggesting that the effect of the functionalization degree on the new PL peak wavelength is small in 2a–2d. Therefore, the differences in the PL wavelength in 2a–2c might be due to the local strain of the SWNTs induced by the cyclic addenda. The effect of the naphthyl unit on the Stokes effect of 2d compared with that of 2c was considered by theoretical calculation which is discussed later.

**Fig. 3:**
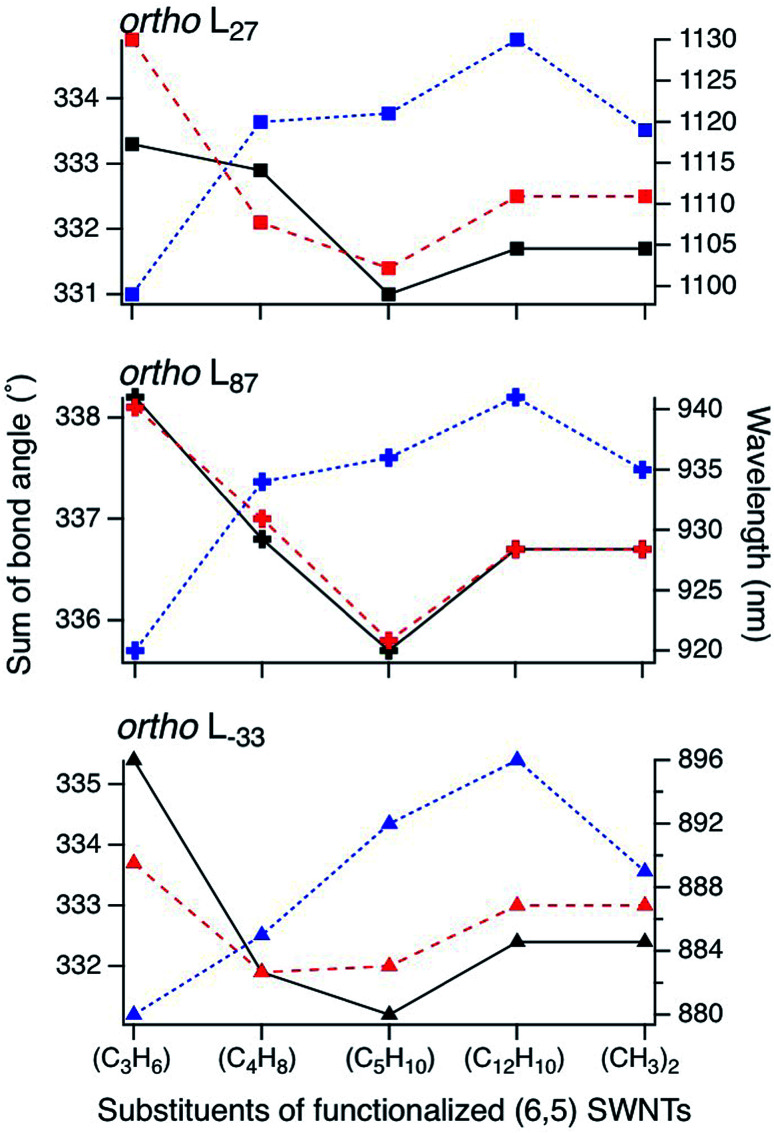
Sum of bond angles at the addition sites in functionalized (6,5) SWNTs (black lines represent the addition position “*a*”, and red lines represent the other addition position in *ortho* adducts). Calculated absorption energy of functionalized (6,5) SWNTs (blue line) using TD-DFT with B3LYP/3-21G. Different orientations in *ortho* adducts are marked in the same manner as in Fig. 2.

To clarify the PL properties of 2, we performed DFT and TD-DFT calculations. We considered six model structures of cyclized adducts with different addition positions. These isomers are distinguished using the abbreviations reported by Htoon and coworkers (Fig. 2a).^21^ The optimized structures of the isomers 2a–2d are shown in Fig. 2c. The calculation results showed that the *ortho* L_−33_ isomer is the most stable isomer, regardless of the functional groups (Fig. 2d). To address the local distortion of 2a–2c, we focus on the bond angles defined in Fig. 2b. The sum of the three bond angles (a_1_ + a_2_ + a_3_) approaches the value of the ideal sp^3^ carbon in the order of SWNT < SWNT-(C_3_H_6_) < SWNT-(C_4_H_8_) < SWNT-(C_5_H_10_) regardless of the addition position (Fig. 3, Tables S2 and S3†). The results indicate that the number of methylene units in the cyclic addenda affects the degree of local strain of the SWNT skeleton.

The absorption energies of *ortho* L_−33_, *ortho* L_87_ and *ortho* L_27_ isomers were calculated using TD-DFT with B3LYP/3-21G. As shown in Fig. 3 and Table 2, the calculated absorption energies strongly depend on their addition position, and increase in the order of SWNT-(C_12_H_10_) < SWNT-(C_5_H_10_), SWNT-(C_4_H_8_) < SWNT-(C_3_H_6_) when compared at the same addition position. Because of the high computational cost of the geometry optimization of the excited state, we calculated the emission energies of *ortho* L_−33_ isomers as selected examples. The calculated emission energies showed a similar tendency to the calculated absorption energy with similar Stokes shifts regardless of the adducts (Table S4†). In order to evaluate the influence of alkyl chain length of acyclic addenda, estimation of absorption energies of SWNT-(C_3_H_6_Br)_2_, SWNT-(C_4_H_8_Br)_2_, H-SWNT-(C_3_H_6_Br), and H-SWNT-(C_4_H_8_Br) was conducted. The calculated absorption energies of SWNT-(C_3_H_6_Br)_2_ and SWNT-(C_4_H_8_Br)_2_ show no significant difference, which is also found in those of H-SWNT-(C_3_H_6_Br) and H-SWNT-(C_4_H_8_Br) (Table S7†). These theoretical calculation results agree well with the experimental results and show that the PL wavelength of SWNTs can be effectively controlled by the alkyl chain length of the cyclic addenda.

**Table 2:** Calculated absorption wavelength of functionalized (6,5) SWNTs using TD-DFT with B3LYP/3-21G

Addition positions	SWNT-(C_3_H_6_)	SWNT-(C_4_H_8_)	SWNT-(C_5_H_10_)	SWNT-(C_12_H_10_)	SWNT-(CH_3_)_2_^a^
*ortho* L_−33_	880	885	892	896	889
*ortho* L_87_	920	934	936	941	935
*ortho* L_27_	1099	1120	1121	1130	1119

a The structure of SWNT in SWNT-(CH_3_)_2_ was taken as the same as that in SWNT-(C_12_H_10_).

To evaluate the effect of the naphthyl unit of 2d, the absorption energy of SWNT-(CH_3_)_2_ was calculated. The structure of SWNT in SWNT-(CH_3_)_2_ was taken to be the same as that in SWNT-(C_12_H_10_) (Fig. 2c). Thus, the distortion of the SWNT in SWNT-(CH_3_)_2_ is identical to that in SWNT-(C_12_H_10_). The absorption energy of SWNT-(CH_3_)_2_ is similar value to that of SWNT-(C_5_H_10_), indicating that the naphthyl unit of 2d contributes to the larger red-shift of 2d than 2c.

**Fig. 4:**
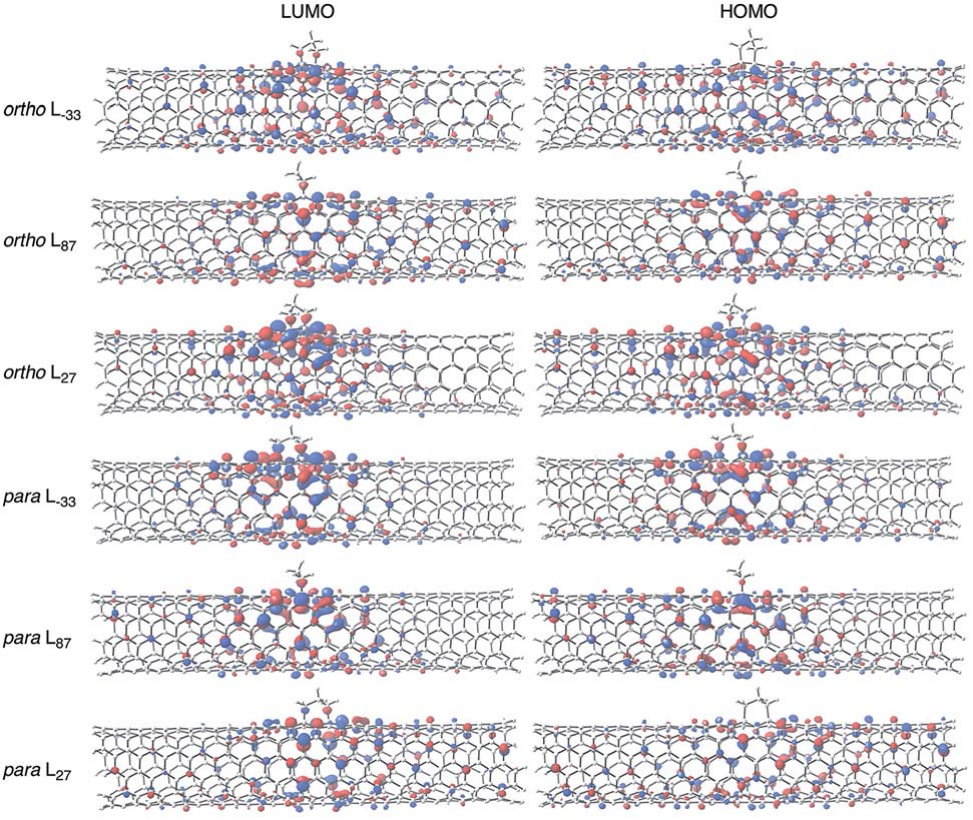
Frontier molecular orbital diagrams of *ortho* and *para* adducts for SWNT-(C_3_H_6_) (B3LYP/6-31G*, isovalue = 0.02).

Focusing on the frontier orbitals, the highest occupied molecular orbital (HOMO) and the lowest unoccupied molecular orbital (LUMO) of the *ortho* L_27_ isomer are the most localized in the *ortho* isomers (Fig. 4, S12 and S13†). In the *para* isomers, HOMO and LUMO orbitals of *para* L_−33_ are the most localized. The degree of localization of the frontier orbital correlates well with the trends in the HOMO–LUMO gap energies, which are shown in Table S5.† This result is consistent with the results of hydrophenylated SWNT isomers reported by Htoon *et al.*^42^

## Conclusions

In conclusion, we studied the PL properties of functionalized SWNTs using dibromoalkanes. After functionalization, new PL peaks were emerged as a dominant peak in the range of 1215–1242 nm depending on the alkyl chain length of the dibromoalkane used. The functionalization degree of the alkylated SWNTs were strongly influenced by the alkyl chain length of the addenda. The DFT calculations showed that the structural changes in the SWNTs increased with increasing alkyl chain length of the cyclic addenda. In addition, theoretical calculations revealed that the naphthyl unit and the length of the alkyl group in the addenda influence the absorption and emission energy of functionalized SWNTs. We expected that these findings will broadly impact for the fundamental understanding of the intrinsic PL properties of SWNTs and will be useful for the development of photonic devices, bioimaging, strain sensing, and other applications.

## Conflicts of interest

There are no conflicts to declare.

## Supplementary Material

RA-009-C9RA02183C-s001
