# Supplementary material for: Influence of local strain caused by cycloaddition on the band gap control of functionalized single-walled carbon nanotubes
Source: RSC Adv. 2019 May 8;9(25):13998–4003. doi: 10.1039/c9ra02183c (PMC9064015; doi:10.1039/c9ra02183c)
Supplement: RA-009-C9RA02183C-s001 [file RA-009-C9RA02183C-s001.pdf]

Electronic Supplementary Material (ESI) for RSC Advances.
This journal is © The Royal Society of Chemistry 2019

Electronic Supplementary Information (ESI)

Influence of local strain caused by cycloaddition on the band gap control of functionalized single-walled carbon nanotubes

Yutaka Maeda,[*a] Kiyonori Kuroda,[a] Haruto Tambo,[a] Hiyori Murakoshi,[a] Yui Konno,[a] Michio Yamada[a], Pei Zhao,[b] Xiang Zhao,[c] Shigeru Nagase,[d] Masahiro Ehara[*b]

[a]Department of Chemistry, Tokyo Gakugei University, Tokyo 184-8501, Japan

[b]Research Center for Computational Science, Institute for Molecular Science, Okazaki, 444-8585, Japan

[c]Institute for Chemical Physics & Department of Chemistry, School of Science, State Key Laboratory of Electrical Insulation and Power Equipment, Xi'an Jiaotong University, Xi'an 710049, China

[d]Fukui Institute for Fundamental Chemistry (FIFC), Kyoto University, Sakyou-ku, Kyoto 606-8103, Japan

p.2 **Fig. S1** (a) Absorption spectra normalized by the local minimum near 775 nm, (b) PL spectra (567 nm excitation), and Raman spectra of SWNTs (Black) and **2a** (Red), **2b** (Yellow), **2c** (Green), and **2d** (Blue) dispersed in D_2O containing 1 wt% SDBS.

p.3 **Fig. S2** Raman spectra of **2a-2d** (561 nm excitation).

p.4 **Fig. S3** E_{11} abs. ratio as a function of D/G (561 nm excitation).

Fig. S4 Contour plots of PL intensity versus excitation and emission wavelengths of **2** after thermal treatment dispersed in D_2O containing 1 wt% SDBS.

p.5 **Fig. S5** TG curve of SWNTs and **2a-2d**. Heating rate:10°C/min. Gas flow rate: 50 mL/min.

Fig. S6 PL, absorption, and Raman spectra of butylated SWNTs having different functionalization degree.

Fig. S7 SEM images of **2a-2d.**

p.6 **Fig. S8** The optimized structures of six adducts for SWNT-(C_3H_6).

Fig. S9 The optimized structures of six adducts for SWNT-(C_4H_8).

Fig. S10 The optimized structures of six adducts for SWNT-(C_5H_{10}).

p.7 **Fig. S11** The optimized structures of six adducts for SWNT-($C_{12}H_{10}$).

Fig. S12 Frontier molecular orbital diagrams of the pristine (6,5) SWNT at the level of B3LYP/6-31G* (isovalue = 0.02).

p.8 **Fig. S13** Frontier molecular orbital diagrams of ortho and para adducts for SWNT-($C_{12}H_{10}$) (B3LYP/6-31G*, isovalue = 0.02).

p.9 **Table S1** Relative energies (in kcal/mol) of functionalized (6,5) SWNTs calculated by DFT with B3LYP/6-31G*.

Table S2 Bond angles of different addition sites in pristine and functionalized (6,5) SWNTs at the level of B3LYP/6-31G*.

p.10 **Table S3** Sum of bond angles at the addition sites in pristine and functionalized (6,5) SWNTs at the level of B3LYP/6-31G*.

Table S4 Calculated absorption and emission wavelengths (in nm) of functionalized (6,5) SWNTs (ortho L_{-33}) using TD-DFT with B3LYP/3-21G.

Table S5 HOMO-LUMO gaps (in eV) of (6.5) SWNT and functionalized (6,5)-SWNTs (B3LYP/6-31G*).

p.11 **Table S6** Total energies (in a.u.) of functionalized (6,5) SWNTs calculated by DFT with B3LYP/6-31G*.

Table S7 Calculated absorption wavelength (in nm) of SWNT-(C_3H_6Br)$_2$, SWNT-(C_4H_8Br)$_2$, H-SWNT-(C_3H_6Br), and H-SWNT-(C_4H_8Br) at the level of B3LYP/3-21G.

Table S8 Weight loss, weight ratio of addenda to SWNTs in functionalized SWNTs, and functional group coverage (FGC).

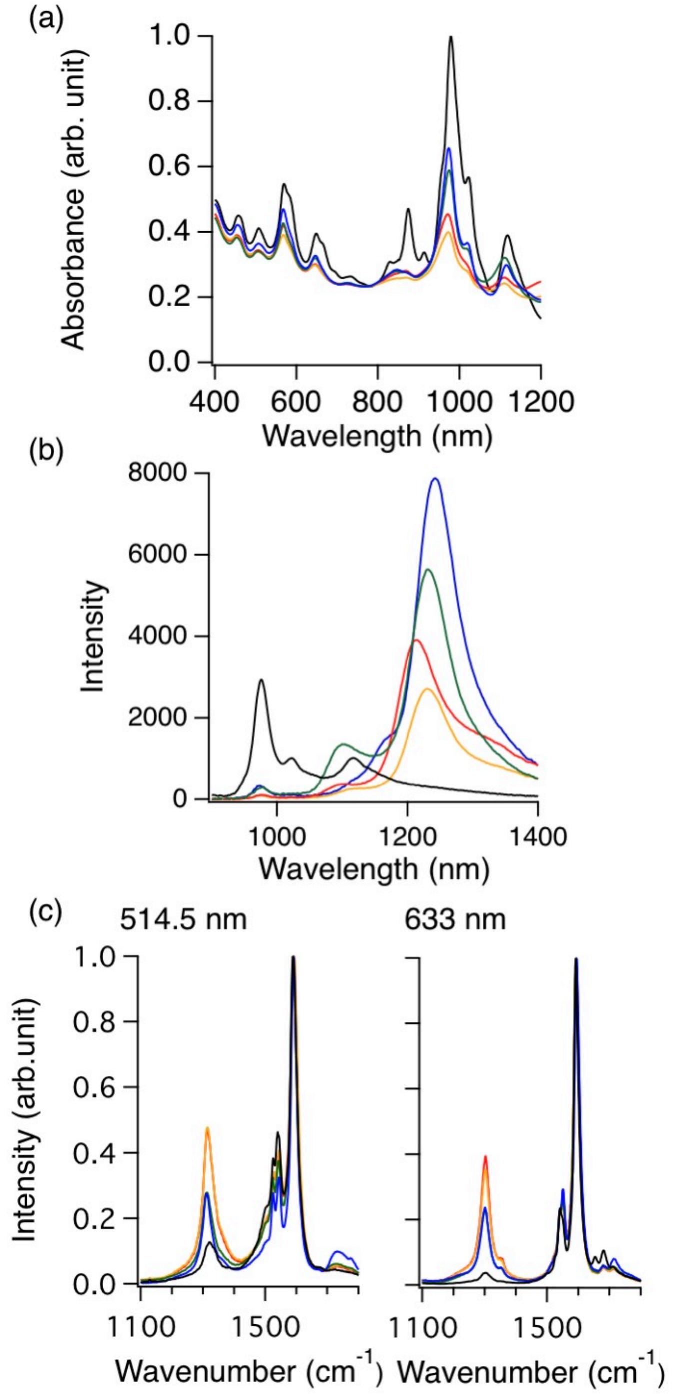

Fig. S1 (a) Absorption spectra normalized by the local minimum near 775 nm, (b) PL spectra (567 nm excitation), and (c) Raman spectra of SWNTs (Black) and **2a** (Red), **2b** (Yellow), **2c** (Green), and **2d** (Blue) dispersed in D_2O containing 1 wt% SDBS.

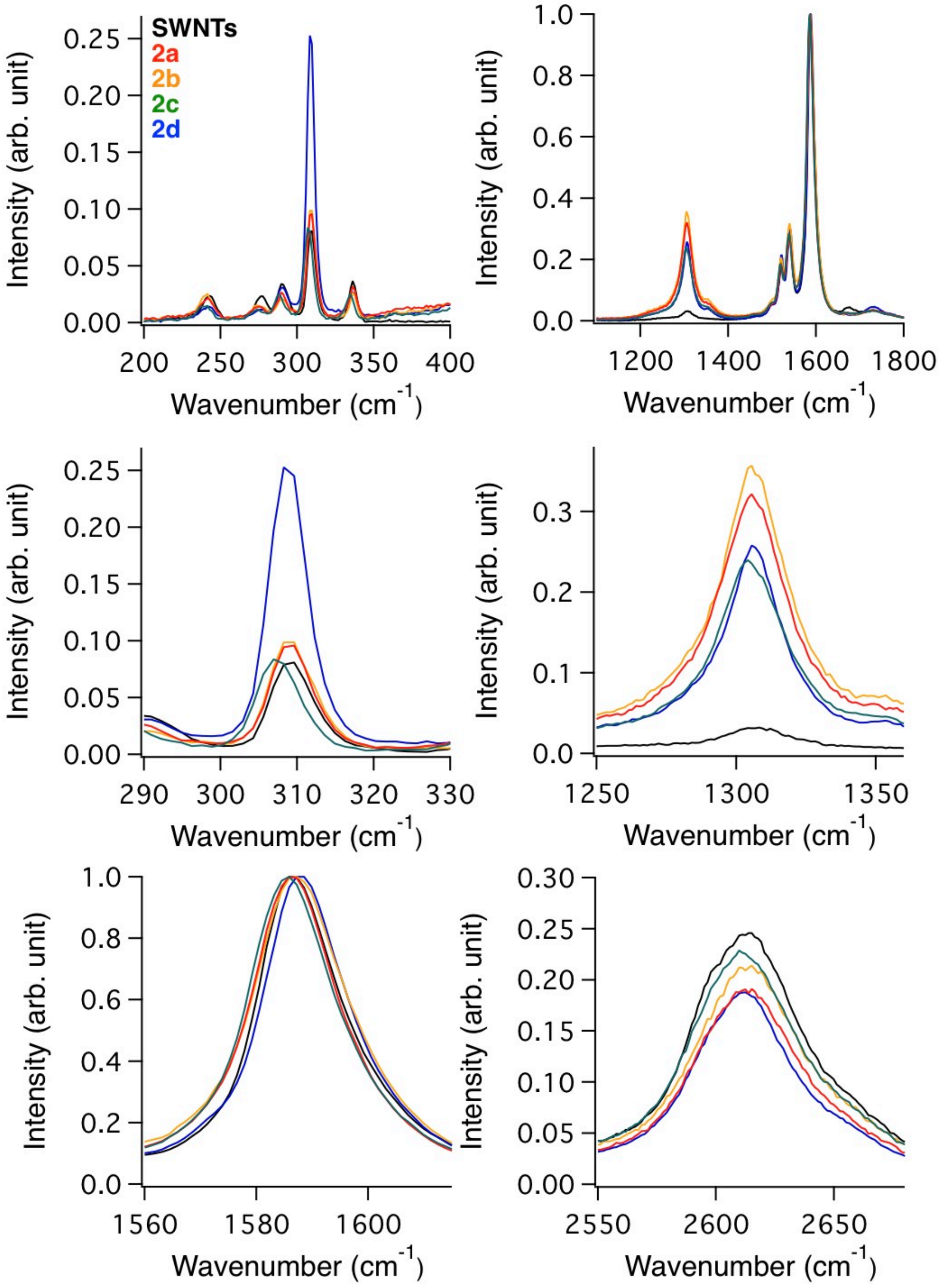

Fig. S2 Raman spectra of SWNTs and **2a**-**2d** (561 nm excitation).

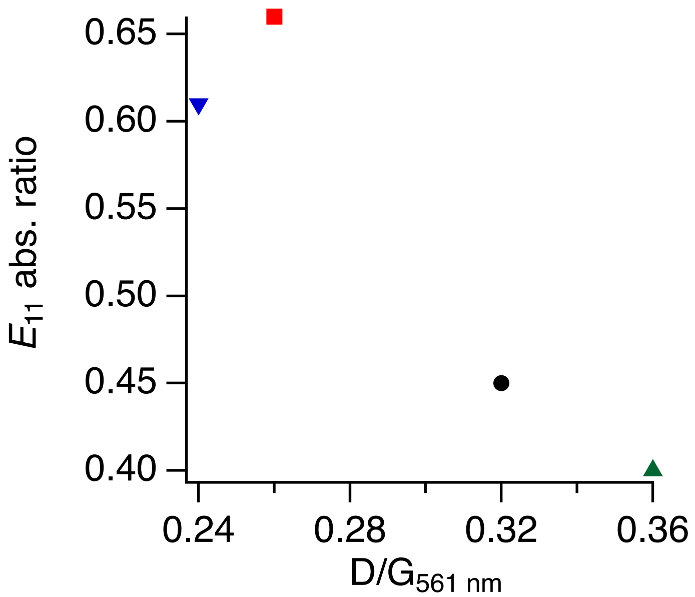

Fig. S3 E_{11} abs. ratio as a function of D/G (561 nm excitation).

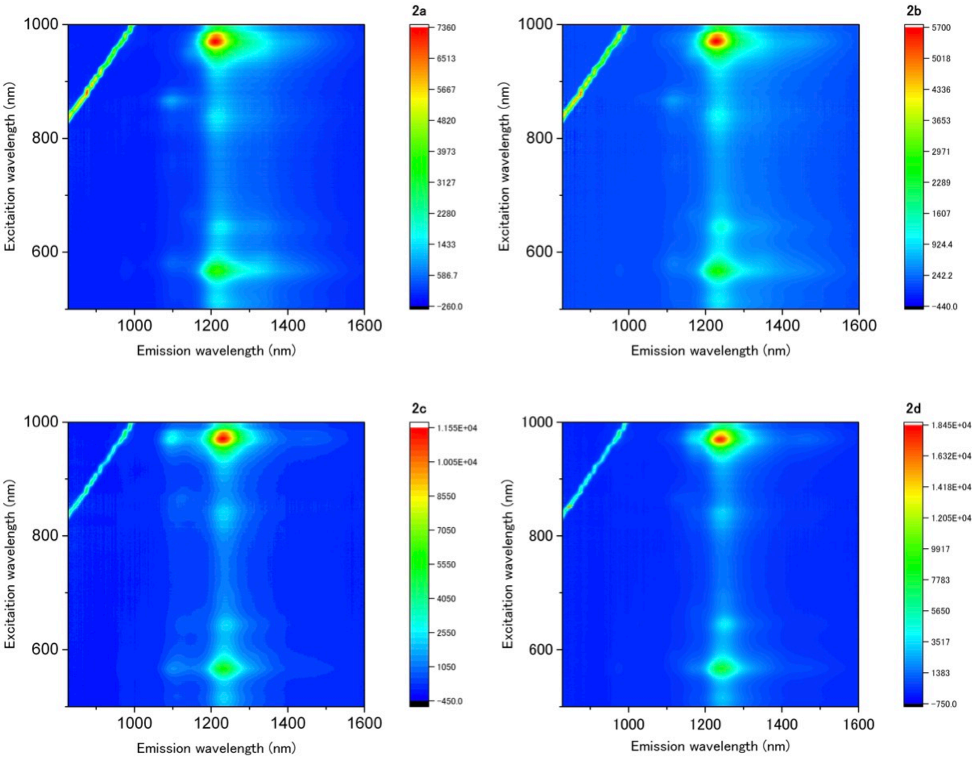

Fig. S4 Contour plots of PL intensity versus excitation and emission wavelengths of **2** dispersed in D_2O containing 1 wt% SDBS.

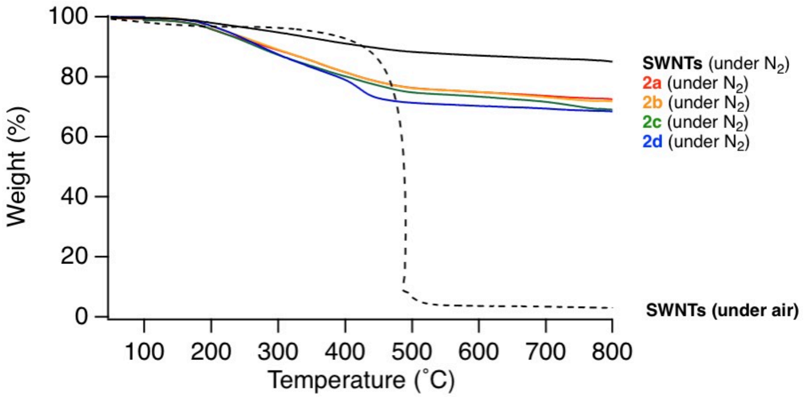

Fig. S5 TG curve of SWNTs and **2a-2d**. Heating rate:10˚C/min. Gas flow rate: 50 mL/min.

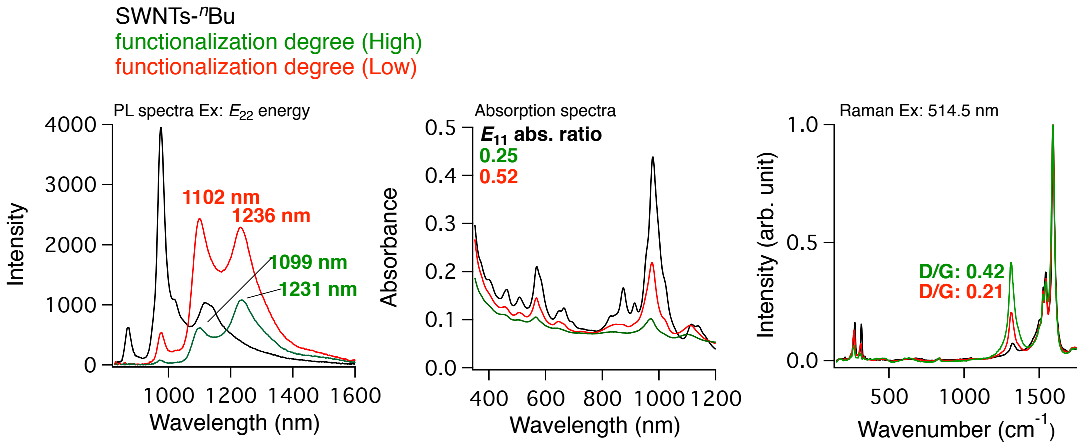

Fig. S6 PL, absorption, and Raman spectra of butylated SWNTs having different functionalization degree.[1]

[1]Y. Maeda, Y. Takehana, J.-S. Dang, M. Suzuki, M. Yamada, S. Nagase, *Chem. Eur. J.* 2017, **23**, 1789-1794.

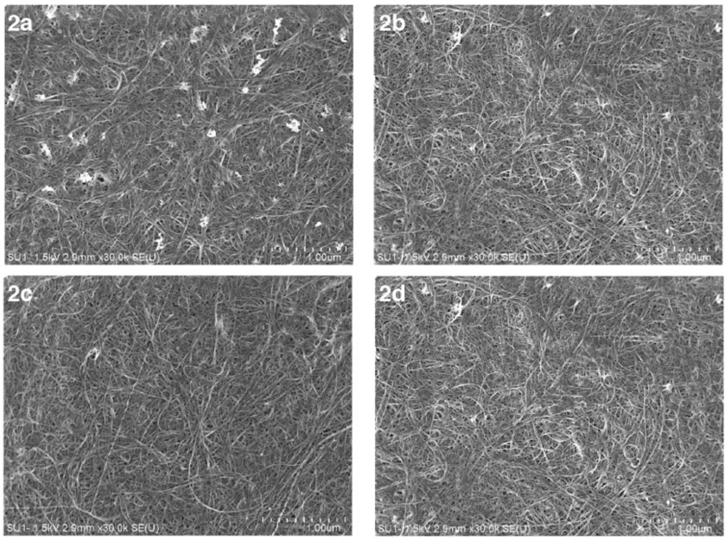

Fig. S7 SEM images of **2a-2d.**

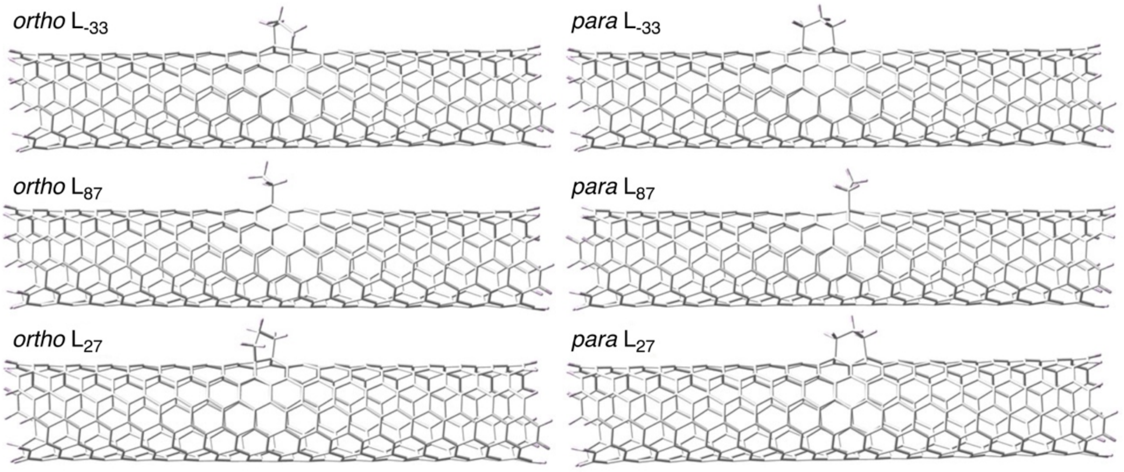

Fig. S8 The optimized structures of six adducts for SWNT-(C_3H_6).

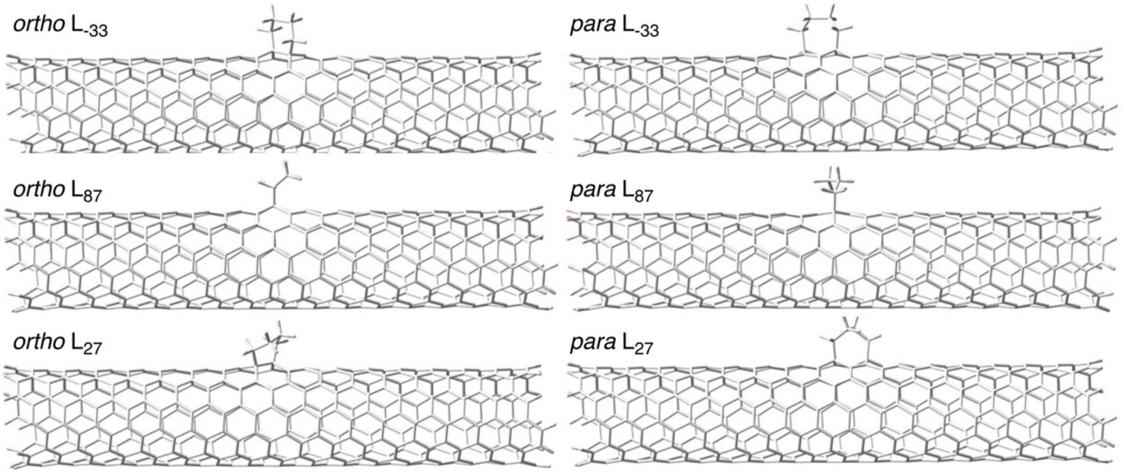

Fig. S9 The optimized structures of six adducts for SWNT-(C_4H_8).

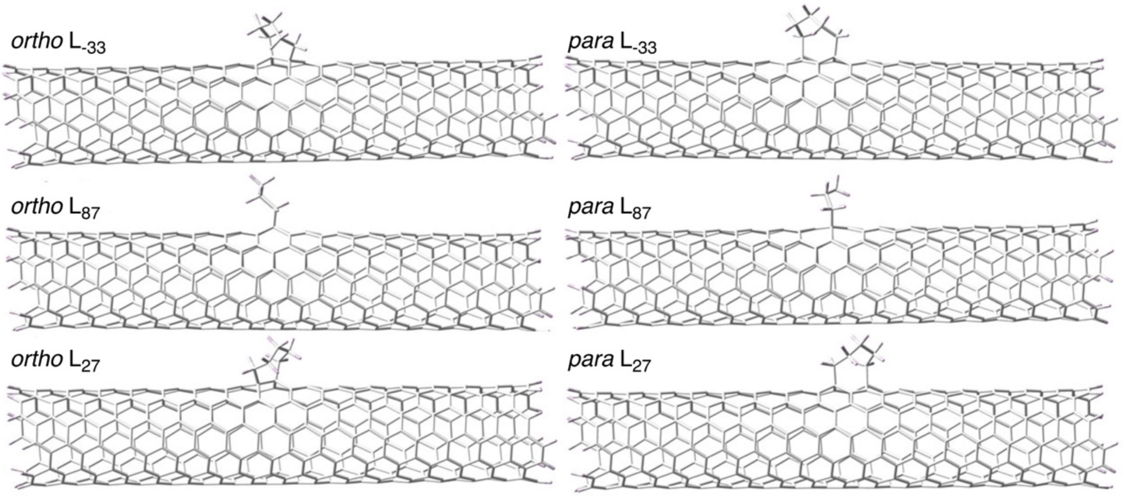

Fig. S10 The optimized structures of six adducts for SWNT-(C_5H_{10}).

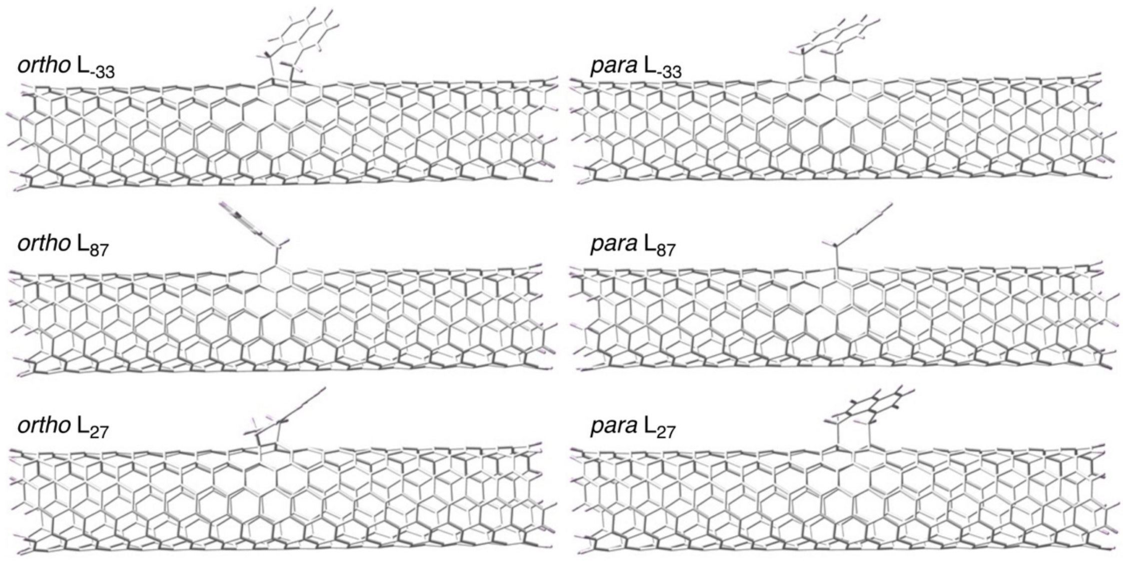

Fig. S11 The optimized structures of six adducts for SWNT-($C_{12}H_{10}$).

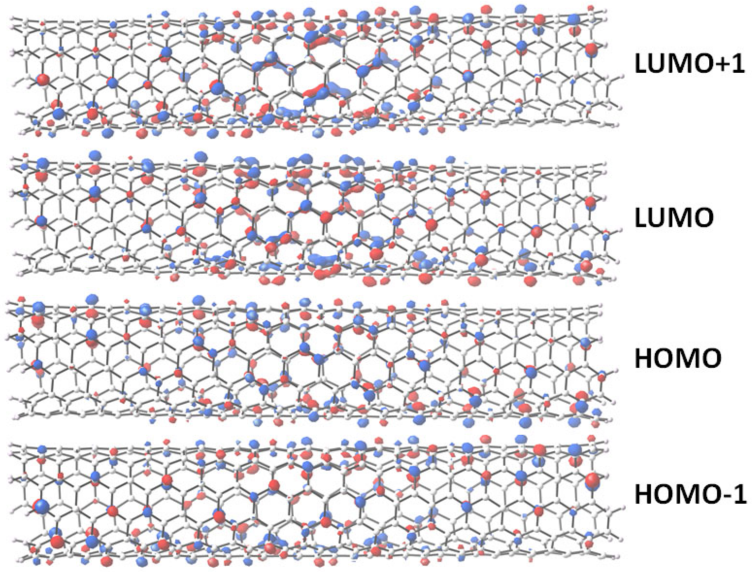

Fig. S12 Frontier molecular orbital diagrams of the pristine (6,5) SWNT at the level of B3LYP/6-31G* (isovalue=0.02).

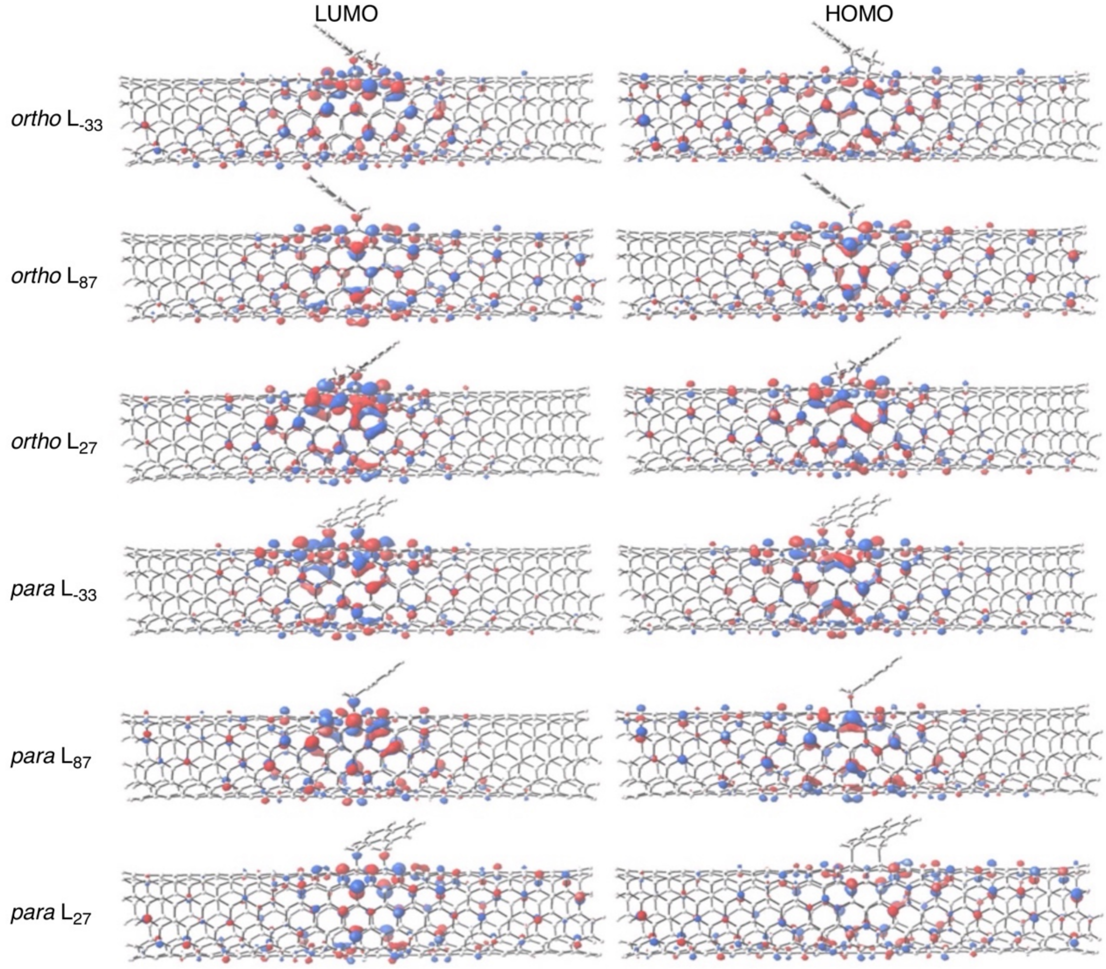

Fig. S13 Frontier molecular orbital diagrams of ortho and para adducts for SWNT-($C_{12}H_{10}$) (B3LYP/6-31G*, isovalue=0.02).

Table S1. Relative energies (in kcal/mol) of functionalized (6,5) SWNTs calculated by DFT with B3LYP/6-31G*.

Carbon position	SWNT-(C_3H_6)	SWNT-(C_4H_8)	SWNT-(C_5H_{10})	SWNT-($C_{12}H_{10}$)
ortho L_{-33}	0	0	0	0
ortho L_{27}	1.9	3.7	2.4	2.4
ortho L_{87}	4.6	7.9	5.8	5.8
para L_{27}	9.6	6.4	11.3	13.2
para L_{-33}	15.8	11.7	14.5	17.5
para L_{87}	46.5	18.2	5.6	39.0

Table S2. Bond angles of different addition sites in pristine and functionalized (6,5) SWNTs at the level of B3LYP/6-31G*.

Addition		SWNT			SWNT-(C_3H_6)			SWNT-(C_4H_8)			SWNT-(C_5H_{10})			SWNT-($C_{12}H_{10}$)		
positions		a_1	a_2	a_3	a_1	a_2	a_3	a_1	a_2	a_3	a_1	a_2	a_3	a_1	a_2	a_3
ortho L_{-33}	*a*	119.1	119.5	118.7	107.6	111.8	116.0	106.4	111.1	114.4	105.6	111.9	113.7	106.3	112.0	114.1
	b	119.1	119.5	118.7	107.2	111.4	115.1	106.4	111.1	114.4	106.1	111.7	114.2	106.5	111.9	114.6
ortho L_{87}	*a*	119.5	118.7	119.1	113.4	112.6	112.2	112.7	112.1	112.0	112.1	111.6	112.0	112.5	111.9	112.3
	d	119.5	118.7	119.1	113.4	112.3	112.4	112.7	112.1	112.2	112.1	112.0	111.7	112.6	112.2	111.9
ortho L_{27}	*a*	118.7	119.1	119.5	106.1	115.4	111.8	105.4	115.0	112.5	104.4	114.5	112.1	105.1	114.5	112.1
	f	118.7	119.1	119.5	106.5	116.2	112.2	105.5	114.5	112.1	105.0	114.7	111.7	105.4	115.0	112.1
para L_{-33}	*a*	119.5	118.7	119.1	112.0	108.2	108.6	112.4	108.2	108.0	112.2	107.2	107.5	112.4	108.3	108.2
	e	119.5	118.7	119.1	112.1	108.2	108.8	112.4	108.2	108.0	112.6	108.0	107.7	112.3	107.9	108.1
para L_{87}	*a*	118.7	119.1	119.5	107.1	107.6	114.3	107.6	108.3	112.6	107.7	108.7	111.7	107.5	107.6	113.0
	g	118.7	119.1	119.5	107.0	107.7	114.4	107.6	108.3	112.6	108.0	108.4	111.7	107.0	108.1	113.0
para L_{27}	*a*	119.1	119.5	118.7	109.0	112.3	107.8	108.8	112.8	107.4	108.1	112.2	106.6	108.7	112.4	107.2
	c	119.1	119.5	118.7	109.1	112.2	107.6	108.8	112.8	107.3	108.7	112.7	106.8	109.0	112.6	107.3

Table S3. Sum of bond angles at the addition sites in pristine and functionalized (6,5) SWNTs at the level of B3LYP/6-31G*.

Addition position		SWNTs	SWNT-(C_3H_6)	SWNT-(C_4H_8)	SWNT-(C_5H_{10})	SWNT-($C_{12}H_{10}$)
ortho L_{-33}	*a*	357.3	335.4	331.9	331.2	332.4
	b	357.3	333.7	331.9	332.0	333.0
ortho L_{87}	*a*	357.3	338.2	336.8	335.7	336.7
	d	357.3	338.1	337.0	335.8	336.7
ortho L_{27}	*a*	357.3	333.3	332.9	331.0	331.7
	f	357.3	334.9	332.1	331.4	332.5

Table S4. Calculated absorption and emission wavelengths (in nm) of functionalized (6,5) SWNTs (*ortho* L_{-33}) using TD-DFT with B3LYP/3-21G.

	SWNT	SWNT-(C_3H_6)	SWNT-(C_4H_8)	SWNT-(C_5H_{10})	SWNT-($C_{12}H_{10}$)	SWNT-($(CH_3)_2$)[a]
Absorption wavelength (calcd.)	790	880	885	892	896	889
Emission wavelength (calcd.)	806	922	927	932	936	930
Stokes shift	16	42	42	40	40	41

[a] The structure of SWNT in SWNT-$(CH_3)_2$ was taken as the same as that in SWNT-($C_{12}H_{10}$).

Table S5. HOMO-LUMO gaps (in eV) of (6.5)-SWNT and functionalized (6,5) SWNTs (B3LYP/6-31G*).

Addition position	SWNT	SWNT-(C_3H_6)	SWNT-(C_4H_8)	SWNT-(C_5H_{10})	SWNT-($C_{12}H_{10}$)
ortho L_{-33}	1.69	1.62	1.61	1.60	1.60
ortho L_{27}	1.69	1.30	1.28	1.28	1.27
ortho L_{87}	1.69	1.55	1.53	1.53	1.53
para L_{27}	1.69	1.66	1.67	1.67	1.66
para L_{-33}	1.69	1.25	1.27	1.26	1.26
para L_{87}	1.69	1.41	1.43	1.43	1.42

Table S6. Total energies (in a.u.) of functionalized (6,5) SWNTs calculated by DFT with B3LYP/6-31G*.

Addition position	SWNT-(C_3H_6)	SWNT-(C_4H_8)	SWNT-(C_5H_{10})	SWNT-($C_{12}H_{10}$)
ortho L_{-33}	-14003.7374286	-14043.0454506	-14082.3472172	-14349.1068842
ortho L_{27}	-14003.7343939	-14043.0395043	-14082.3433409	-14349.1030330
ortho L_{87}	-14003.7301413	-14043.0329049	-14082.3379451	-14349.0977008
para L_{27}	-14003.7221869	-14043.0351775	-14082.3291672	-14349.0859153
para L_{-33}	-14003.7123257	-14043.0268116	-14082.3240413	-14349.0790522
para L_{87}	-14003.6632968	-14043.0164373	-14082.3383659	-14349.0446623

Table S7. Calculated absorption wavelength (in nm) of SWNT-($C_3H_6Br)_2$, SWNT-($C_4H_8Br)_2$, H-SWNT-(C_3H_6Br), and H-SWNT-(C_4H_8Br) at the level of B3LYP/3-21G.

Addition position	Functionalized SWNTs	Absorption wavelength	Oscillator strength
para L_{87}	SWNT-($C_3H_6Br)_2$	1005	0.8951
	SWNT-($C_4H_6Br)_2$	1003	0.9049
ortho L_{-33}	H-SWNT-C_3H_6Br	890	0.5075
	H-SWNT-C_4H_6Br	889	0.5123

Table S8. Weight loss, weight ratio of addenda to SWNTs in functionalized SWNTs, and functional group coverage (FGC).

	**2a**	**2b**	**2c**	**2d**
Weight ratio of addenda to SWNTs[1,2]	15.1 : 84.9	16.0 : 84.0	19.3 : 80.7	19.9 : 80.1
FGC[3] (number of C / one substituent)	19.7	24.6	24.5	51.5

1 Ratio of SWNTs and catalyst (wt%) was 97.0 : 3.0 estimated by TGA under air.

2 wt% of addenda was corrected by wt loss of SWNTs under the same condition.

3 F. G. Brunetti, M. A. Herrero, J. D. M. Muñoz, A. Díaz-Ortiz, J. Alfonsi, M. Meneghetti, Prato, E. J. Vázquez, E. *J. Am. Chem. Soc.* 2008, **130**, 8094-8100.
